# Demographic and work-related factors associated with burnout, resilience, and quality of life among healthcare workers during the COVID-19 pandemic: A cross sectional study from Malaysia

**DOI:** 10.3389/fpubh.2022.1021495

**Published:** 2022-12-16

**Authors:** Roy Rillera Marzo, Mohamed ElSherif, Muhd Siv Azhar Merican Bin Abdullah, Hui Zhu Thew, Collins Chong, Shean Yih Soh, Ching Sin Siau, Shekhar Chauhan, Yulan Lin

**Affiliations:** ^1^International Medical School, Management and Science University, Shah Alam, Selangor, Malaysia; ^2^Global Public Health, Jeffrey Cheah School of Medicine and Health Sciences, Monash University Malaysia, Subang Jaya, Selangor, Malaysia; ^3^Department of Epidemiology and Population Health, Faculty of Health Sciences, American University of Beirut, Beirut, Lebanon; ^4^Department of Community Health, Faculty of Medicine, Universiti Kebangsaan Malaysia, Kuala Lumpur, Malaysia; ^5^Department of Family Medicine, Faculty of Medicine and Health Sciences, Universiti Putra Malaysia, Serdang, Selangor, Malaysia; ^6^Department of Anaesthesia and Intensive Care, Faculty of Medicine and Health Sciences, Universiti Putra Malaysia, Serdang, Selangor, Malaysia; ^7^Department of Psychiatry, Faculty of Medicine and Health Sciences, Universiti Putra Malaysia, Serdang, Selangor, Malaysia; ^8^Centre for Community Health Studies, Faculty of Health Sciences, Universiti Kebangsaan Malaysia, Kuala Lumpur, Malaysia; ^9^Department of Family and Generations, International Institute for Population Sciences, Mumbai, India; ^10^Department of Epidemiology and Health Statistics, School of Public Health, Fujian Medical University, Fuzhou, China

**Keywords:** burnout, resilience, quality of life, influencing factors, healthcare workers, Malaysia

## Abstract

**Introduction:**

The healthcare setting is a stressful and demanding work environment, and healthcare workers face a continuous expansion of their job roles and responsibilities. Past studies have shown that factors affecting burnout, resilience, and quality of life among healthcare workers merit further research, as there were inconsistent findings, especially with regards to the influence of demographic and work-related factors. Therefore, this study aims to determine whether demographic and work-related factors are associated with burnout, resilience, and quality of life among healthcare workers.

**Method:**

This cross-sectional study was conducted between February 15, 2022 and March 15, 2022, among 394 healthcare workers from Putrajaya and Selangor hospitals, Malaysia. Maslach Burnout Inventory, World Health Organization Quality of Life-BREF 26 inventory, and Brief Resilience Scale were utilized to capture information on burnout, quality of life, and resilience, respectively.

**Results:**

The mean score of physical health of participants who work more than 10 h (11.38) is lower than participants who work from 8 to 10 h (13.00) and participants who work 7 h daily (13.03), *p*-value < 0.001. Similarly, the mean score of psychological health of participants who work more than 10 h (12.35) is lower than participants who work from 8 to 10 h (13.72) and participants who work 7 h daily (13.68), *p*-value = 0.001. Higher income levels were associated with high resilience and quality of life.

**Conclusion:**

It is imperative that healthcare practitioners and policy makers adopt and implement interventions to promote a healthy workplace environment, address ethical concerns, and prevent burnout among healthcare workers during the COVID-19 pandemic. Managing the issue of long working hours could possibly result in improved resilience, burnout, and quality of life among healthcare workers. Despite this study able to tickle out some policy specific areas where interventions are needed, identifying effective solutions and evaluating their efficiency will require larger and interventional studies.

## Introduction

The healthcare setting is a stressful and demanding work environment, and healthcare workers (HCWs) face a continuous expansion of their job roles and responsibilities, such as increasing bureaucratic tasks and computerization of the healthcare system (1, 2). With the advent of the coronavirus disease 2019 (COVID-19) pandemic, a worldwide medical emergency, additional stress was put upon the healthcare system, resulting in high levels of psychological distress and burnout among HCWs (3–7). General population across countries is also affected by COVID-19 pandemic in terms of their health (8–10). This study explored burnout, resilience, and quality of life (QoL) among HCWs in Malaysia.

Burnout refers to a state of exhaustion resulting from prolonged stress, and is typified by three syndromes, including emotional exhaustion (EE), depersonalization (DP), and personal accomplishment (PA) (11). An individual who experiences burnout would typically report physical and emotional overextension, feelings of cynicism and callousness toward his/her work, and a worse level of professional efficacy (11). In the latest version of the International Classification of Diseases-11 (12), burnout has now been redefined as a workplace phenomenon involving the three syndromes described above, rather than as a result of difficulty in the management broad life circumstances. The prevalence of burnout in the healthcare setting has been high, both before and during the COVID-19 pandemic (13, 14). During the COVID-19 pandemic, the level of burnout among HCWs was expected to be higher due to longer working hours, sleep deprivation, and the need to adhere to preventive measures against the virus (15, 16). The pooled prevalence of burnout found in thirty observational studies was 52.0%, with DP being the highest syndrome (52.0%), followed by EE (51.0%), and low PA (28.0%) (17).

Burnout is an important area for further investigation, as burnout syndrome among HCWs has been shown to have service implications such as worse patient safety (18). A number of factors are associated with burnout among HCWs. Systematic reviews showed that job stress, time pressure, high workload, long working hours, low job satisfaction, and low organizational support were factors associated with burnout (19, 20). Demographic factors associated with burnout were younger age, female sex, and marital status (20). A study in Malaysia during the COVID-19 pandemic showed that workload, uncertainties caused by the pandemic, challenging work-family balance, and stretched workplace relationships influenced burnout among HCWs (21).

As HCWs are among the professionals most affected by the COVID-19 pandemic, it is important to study how psychological resilience helped them to cope (22). Resilience refers to an individual's ability to adapt to and rebound from negative workplace stresses such as conflict, failure, and uncertainty (23, 24). Inculcating resilience involves developing coping strategies in order to pre-empt reactions to stressful situations (25). A study showed that nurses with a higher education (postgraduate degree vs. bachelor's degree) exhibited higher resilience against developing posttraumatic stress (26). Demographic factors were inconsistently associated with resilience. For example, systematic reviews among doctors and nurses found differing results in the significance of age, education level, income, marital status, work experience, and job status on resilience (27, 28). Other factors, such as burnout and high psychological distress, however, were consistently associated with lower resilience among HCWs (27, 28). During the COVID-19 pandemic, a study among Portuguese HCWs showed that psychological resilience had a mediating effect in the relationship between depression and burnout (29).

The World Health Organization (WHO) defines QoL as “the individual's perception of their position in life in the context of the culture and value systems in which they live and in relation to their goals, expectations, standards and concerns” (30). Apart from biomedical outcomes, QoL has been increasingly used as a yardstick to measure wellbeing in health research (31). However, while it has been used extensively among patients, there is less research on HCW's QoL. A study in Saudi Arabia showed that a high proportion of nurses reported good QoL (32). Another study in India during the COVID-19 pandemic showed that low QoL was prevalent among HCWs, at 45.0% (33). Factors associated with QoL among HCWs were not consistent across studies. For example, a study among Iranian nurses found that work experience, gender, job position and group of patients treated were unrelated with QoL (34). However, another study from Saudi Arabia showed that demographic and work factors such as age, marital status, having children, income, education, working experience, and department were associated with QoL (32).

Past studies have shown that factors affecting burnout, resilience, and QoL among HCWs merit further research, as there were inconsistent findings, especially with regards to the influence of demographic and work-related factors. During the COVID-19 pandemic, HCWs are also exposed to unique factors that may affect their levels of burnout, resilience, and QoL. COVID-19 infection status may affect an individual in all aspects of their lives. For example, an individual will experience negative physical symptoms, psychological fear, and social isolation when infected by the virus (35), thus affecting their QoL. Being infected by the COVID-19 will also exacerbate burnout symptoms (36). Therefore, it is important to study the association between COVID-19 infection status and HCWs' burnout, resilience, and QoL.

Due to the medical crisis of the COVID-19 pandemic, prolonging into an endemic, identification of factors affecting burnout, resilience, and QoL among HCWs could help to focus on specific points for intervention in order to build a strong healthcare workforce. Therefore, this study aims to determine whether gender, specialty, age, education level, income level, work duration, duration of socialization, and COVID-19 infection status are associated with burnout, resilience, and QoL among HCWs.

## Materials and methods

### Study population and sampling

This cross-sectional study was conducted between February 15, 2022 and March 15, 2022 to evaluate the level of burnout, QoL, and resilience among HCWs from Putrajaya and Selangor hospitals of Malaysia. The study was conducted during the fifth wave fuelled by the Omicron variant that led to maximum daily cases in February and March 2022 (37, 38), but is marked by lower numbers of hospitalizations and deaths than during the spread of the Delta variant (38). As of March 2022, the BA.2 Omicron sub-variant was projected to be the dominant strain in the country (39). The country's vaccination programme, which commenced in late February 2021 (40), has fully inoculated over 80% of the population and 97% of adults as of April 24, 2022 (41). On February 13, 2022, the total number of cases in Malaysia exceeded the 3 million mark, reaching 3,040,235 (41). By February 24, 2022, the total number of recoveries had reached the 3 million mark, reaching 3,018,172 (41).

The study used a convenience sampling method for recruitment. The online survey was disseminated *via* various social media platforms such as Instagram, Twitter, LinkedIn, Facebook, WhatsApp, and Telegram. The target population was adult Malaysian HCWs aged 18 years old and above. We invited Malaysian assistant medical officers, doctors, health inspectors, hospital food preparation personnel, medical laboratory technologists, nurses, paramedics, pharmacists, physicians, physiotherapists, dieticians, therapists, psychologists, counselors, radiographers, and social workers from public and private healthcare services to enroll in this study. All respondents were informed that their participation was anonymous and voluntary at the beginning of the survey. Consent was implied if the participants started answering the questionnaire. This research complied with the tenets of the Declaration of Helsinki.

Cochran's formula was used to calculate the minimum recommended sampling size (42). The minimal sample size required for this study with a confidence level of 95%, ± 5% precision and 0.5 estimated proportion was 385 study participants. A total of 394 completed responses were collected. The Institutional Review Board granted approval and the requirement for written informed consent was waived based on the recognition that answering the survey instrument implied consent. Participation was voluntary and anonymity was assured. All personal information was kept confidential. Furthermore, researchers analyzed only de-identified data.

### Study instruments

#### Sociodemographic and work-related characteristics

The data collection instrument comprised of five parts. The first part of the tool asked questions pertaining sociodemographic and work-related characteristics. The choice of variables was informed by the available literature and inputs from the investigators. Participants were requested to indicate their age, gender, marital status, specialty, educational level, income, number of family members, job title, place of work, years of experience, hours of working, and socialization time per week. This section also asked whether the respondent had been attending COVID-19 patient directly, had been infected with COVID-19, and their willingness to have another COVID-19 vaccine's booster doses in the future.

#### Maslach burnout inventory questionnaire

The second part of the study tool was a translated version of Maslach burnout inventory (MBI) (11). To limit the study to burnout related to COVID-19, the phrase “due to COVID-19” was added to each item. MBI is an internationally recognized, validated, self-report questionnaire for measuring the severity of workplace burnout, using the three dimensions of EE, DP, and PA. The questionnaire has 22 items and each item is answered on a seven-point Likert scale. This tool has been extensively used in many studies in different parts of the world and the Malaysian translation has also been validated previously (43, 44).

Burnout is expressed by scores of each of the three MBI subscales, with a high score meaning a high level of burnout. Each subscale score is calculated by adding up all scores of all items in that subscale, with the notion that the items on PA domain are reversely scored (11). Scores range from 0 to 54 for EE, from 0 to 30 for DP, and from 0 to 48 for PA subscale. Scales are scored such that higher scores indicate more of each construct. Higher scores on the EE and DP subscales indicate a higher burnout symptom burden; lower scores on the PA subscale indicate a higher burnout symptom burden. The standard cut-off values were used to define low, moderate, and high levels in each dimension (11). The Cronbach's alpha was 0.86.

#### WHO quality of life-BREF 26

The WHOQOL-BREF is a 26-item instrument consisting of four domains: physical health (seven items), psychological health (six items), social relationships (three items), and environmental health (eight items); it also contains QOL and general health items. Each individual item of the WHOQOL-BREF is scored from 1 to 5 on a response scale, which is stipulated as a five-point ordinal scale (45).

The physical health domain questions are based on daily activities, medical aid, energy, mobility, the extent of pain, sleeping pattern, and working capacity. The psychological domain focuses on participants' personal beliefs, positive and negative feelings, self-esteem, body image, thinking, and learning capabilities. The social relationships domain explores the respondent's overall satisfaction with their personal and social life. Lastly, the environment domain comprises questions about safety and security, contentment with one's property and physical surroundings, finances (does one have enough money to satisfy one's requirements), access to the necessary care, information, and transport. Moreover, the questionnaire has two specific questions regarding participants' opinions regarding their overall QoL and health. We used the Bahasa melayu validated version of the original WHOQOL-BREF questionnaire (46, 47). The Cronbach's alpha was 0.89.

#### Brief Resilience Scale

The last section is the Brief Resilience Scale (BRS) questionnaire to assess the perceived ability to bounce back or recover from stress (48). The scale was developed to assess a unitary construct of resilience, including both positively and negatively worded items.

The BRS has six items presented in Table 1. Items 1, 3, and 5 are positively worded, and items 2, 4, and 6 are negatively worded. The BRS is scored by reverse coding items 2, 4, and 6 and finding the mean of the six items. The following instructions are used to administer the scale: “Please indicate the extent to which you agree with each of the following statements by using the following scale: 1 = strongly disagree, 2 = disagree, 3 = neutral, 4 = agree, 5 = strongly agree.” The possible score range on the BRS is from 1 (low resilience) to 5 (high resilience). It composes of 6 questions with a score on interpretation 1.00–2.99 as low resilience, 3.00–4.30 as normal resilience and lastly 4.31–5.00 as high resilience. The Cronbach's alpha was 0.76.

**Table 1:** Socio-demographics of participants (*N* = 394).

	***N***	**%**
**Gender**		
Male	51	12.9
Female	343	87.1
**Age**		
Less than 25 years	81	20.6
From 25–35 years	170	43.1
From 35–55 years	123	31.2
More than 55 years	20	5.1
**Specialty**		
Doctor	55	14.0
Nurse	248	62.9
Others	91	23.1
**Income level**		
≤ RM 4,850	282	71.6
RM 4,850 – RM 10,959	100	25.4
≥ RM 10,959	12	3.0
**Education background**		
Primary	30	7.6
Secondary	178	45.2
Tertiary	186	47.2
**Working experiences**		
No experience	28	7.1
Less than 5 years	125	31.7
From 5–10 years	92	23.4
More than 10 years	149	37.8
**Working duration**		
Less than 7 h	4	1.0
7 h daily	117	29.7
From 8 to 10 h daily	216	54.8
More than 10 h	57	14.5
**Family members**		
Less than 5	152	38.6
5–10	220	55.8
More than 10	22	5.6
**Socializing duration**		
No time	12	3.0
Less than 5 h	95	24.1
From 5 to 10 h	129	32.7
More than 10 h	158	40.1
**Dealing with COVID-19**		
No	205	52.0
Yes	189	48.0
**Infected with COVID-19**		
No	213	54.1
Yes	181	45.9
**COVID-19 vaccine booster**		
Very unlikely	32	8.1
Unlikely	63	16.0
Somewhat unlikely	58	14.7
Somewhat likely	79	20.1
Likely	101	25.6
Very likely	61	15.5

### Data analysis

At first, normality of the data was checked. Descriptive statistics are presented in the form of numbers and percentages for the categorical variables. Mean and standard deviation (SD) are reported for numerical variables. Chi square and exact test were used to compare categorical variables (burnout, resilience levels) across different variables. The independent samples *t*-test and one way ANOVA test were used to compare numerical variables (quality of life) across different variables. IBM SPSS 28 for windows software was used for the analysis, and a *P*-value < 0.05 is considered statistically significant.

### Ethic statement

The study was designed and conducted in line with the declaration of Helsinki (49) and was approved by the Ethics Committee of Management and Science University (Ethics Code: MSU-RMC-02/FR01/09/L1/085). Respondents were informed that their participation was voluntary, and written consent was implied on the completion of the questionnaire. All participants were aged 18 years or older.

## Results

A total of 394 respondents were included in this study. About 87.1% of the participants were females. Age of 43.1% of the participants ranged from 25 to 35 years, 31.2% from 36 to 55 years, 20.6% were < 25 years, and 5.1% were more than 55 years. About 62.9% of participants were nurses, 14% were doctors, and the remaining were from other specialties in the medical field (Table 1).

As depicted in Table 2, burnout, resilience, and QoL levels showed no statistically significant differences between males and females.

**Table 2:** Differences in burnout, quality of life, and resilience levels based on gender.

		**Male**		**Female**		***P*-value**
		***N***	**%**	***N***	**%**	
Burnout	Occupational exhaustion					0.176
	Low degree	1	2.90%	34	97.10%	
	Moderate degree	28	14.10%	171	85.90%	
	High degree	22	13.80%	138	86.30%	
	Degree of depersonalization					0.901
	Low degree	0	0.00%	5	100.00%	
	Moderate degree	4	10.50%	34	89.50%	
	High degree	47	13.40%	304	86.60%	
	Degree of personal accomplishment					0.159
	Low degree	34	11.30%	267	88.70%	
	Moderate degree	12	20.30%	47	79.70%	
	High degree	5	14.70%	29	85.30%	
Quality of life (mean, SD)	Physical health	12.41	2.66	12.83	2.28	0.230
	Psychological health	13.07	2.88	13.59	2.45	0.173
	Level of social	14.12	3.99	14.18	3.26	0.909
	Level of environment	13.93	2.86	14.27	2.58	0.396
Resilience	Low	10	10.6%	84	89.4%	0.729
	Normal	40	13.6%	254	86.4%	
	High	1	16.7%	5	83.3%	

Burnout showed no statistically significant differences between different specialties among the HCWs (Table 3). Levels of QoL and resilience showed statistically significant differences between different specialties. The mean score of physical health of nurses is higher than that of doctors (13.13 vs. 11.79, *p*-value ≤ 0.001). The mean score of psychological health of nurses is higher than doctors (13.78 vs. 12.70, *p*-value = 0.009). The mean score of social relationships of nurses is higher than doctors (14.61 vs. 13.24, *p*-value = 0.003). Of all the participants with low resilience, 19.1% of participants were doctors, 53.2% were nurses, and 27.7% were from other professions. Similarly, about 11.9% of participants who have normal resilience were doctors, 66.7% were nurses, and 21.4% were from other specialty (*p*-value = 0.038).

**Table 3:** Differences in burnout, quality of life, and resilience levels based on specialty.

		**Doctor**		**Nurse**		**Others**		***P*-value**
		***N***	**%**	***N***	**%**	***N***	**%**	
Burnout	Occupational exhaustion							0.104
	Low degree	2	5.70%	24	68.60%	9	25.70%	
	Moderate degree	22	11.10%	132	66.30%	45	22.60%	
	High degree	31	19.40%	92	57.50%	37	23.10%	
	Degree of depersonalization							0.099
	Low degree	0	0.00%	5	100.00%	0	0.00%	
	Moderate degree	1	10.50%	28	73.70%	9	23.70%	
	High degree	54	13.40%	215	61.30%	82	23.40%	
	Degree of personal accomplishment							0.077
	Low degree	37	12.30%	197	65.40%	67	22.30%	
	Moderate degree	14	23.70%	28	47.50%	17	28.80%	
	High degree	4	11.80%	23	67.60%	7	20.60%	
Quality of life (mean, SD)	Physical health	11.79	1.72	13.13	2.36	12.42	2.37	≤ 0.001
	Psychological health	12.70	2.17	13.78	2.48	13.29	2.69	0.009
	Level of social	13.24	3.08	14.61	3.30	13.57	3.48	0.003
	Level of environment	13.99	2.17	14.46	2.71	13.71	2.57	0.049
Resilience	Low	18	19.1%	50	53.2%	26	27.7%	0.038
	Normal	35	11.9%	196	66.7%	63	21.4%	
	High	2	33.3%	2	33.3%	2	33.3%	

Burnout and resilience levels showed no statistically significant differences between different age groups (Table 4). The only variable that showed statistically significant differences between different age groups is the level of QoL. The mean score of physical health of participants from 25 to 36 years old (12.25) was lower than participants <25 years old (13.09) and participants from 36 to 55 years old (13.31; *p*-value < 0.001). Table 5 shows that burnout and resilience levels had no statistically significant differences between different levels of education. The only variable that showed statistically significant differences between different education levels was level of QoL. The mean score of physical health of participants who had secondary education was higher than participants who had tertiary education (13.12 vs. 12.44, *p*-value = 0.020). The mean score of psychological health participants who had secondary education was higher than participants who had tertiary education (13.91 vs. 13.26, *p*-value = 0.013). Table 6 depicts that burnout showed no statistically significant differences between different levels of income. The variables that showed statistically significant differences between different levels of income were levels of QoL and resilience. The mean score of environment level of participants who took < RM 4,850 was lower than participants who took between RM 4,850 and RM 10,959 and participants who took more than RM 10,959 per month (*p*-value < 0.001). About 16.7% of participants who had high resilience took <RM 4,850, 33.3% took RM 4,850 to RM 10,959, and 50.0% took more than RM 10,959 per month (*p*-value = 0.001).

**Table 4:** Differences in burnout, quality of life, and resilience levels based on age.

		**Less than 25 years**		**From 25 to 35 years**		**From 36 to 55 years**		**More than 55 years**		***P*-value**
		***N***	**%**	***N***	**%**	***N***	**%**	***N***	**%**	
Burnout	Occupational exhaustion									0.607
	Low degree	6	17.10%	13	37.10%	14	40.00%	2	5.70%	
	Moderate degree	44	22.10%	79	39.70%	65	32.70%	11	5.50%	
	High degree	31	19.40%	78	48.80%	44	27.50%	7	4.40%	
	Degree of depersonalization									0.700
	Low degree	1	20.00%	1	20.00%	3	60.00%	0	0.00%	
	Moderate degree	7	18.40%	14	36.80%	15	39.50%	2	5.30%	
	High degree	73	20.80%	155	44.20%	105	29.90%	18	5.10%	
	Degree of personal accomplishment									0.133
	Low degree	68	22.60%	119	39.50%	96	31.90%	18	6.00%	
	Moderate degree	9	15.30%	34	57.60%	15	25.40%	1	1.70%	
	High degree	4	11.80%	17	50.00%	12	35.30%	1	2.90%	
Quality of life (mean, SD)	Physical health	13.09	2.59	12.25	2.42	13.31	1.91	12.80	2.01	≤ 0.001
	Psychological health	13.64	2.86	13.05	2.53	14.01	2.18	14.03	2.21	0.009
	Level of social	13.53	3.34	13.96	3.37	14.82	3.30	14.67	3.09	0.034
	Level of environment	13.85	2.61	13.69	2.67	14.98	2.28	15.63	2.71	≤ 0.001
Resilience	Low	20	21.3%	47	50.0%	26	27.7%	1	1.1%	< 0.001
	Normal	61	20.7%	122	41.5%	96	32.7%	15	5.1%	
	High	0	0.0%	1	16.7%	1	16.7%	4	66.7%	

**Table 5:** Differences in burnout, quality of life, and resilience levels based on education level.

		**Primary education**		**Secondary education**		**Tertiary education**		***P*-value**
		***N***	**%**	***N***	**%**	***N***	**%**	
Burnout	Occupational exhaustion							0.517
	Low degree	4	11.40%	18	51.40%	13	37.10%	
	Moderate degree	13	6.50%	94	47.20%	92	46.20%	
	High degree	13	8.10%	66	41.30%	81	50.60%	
	Degree of depersonalization							0.23
	Low degree	1	20.00%	2	40.00%	2	40.00%	
	Moderate degree	2	5.30%	23	60.50%	13	34.20%	
	High degree	27	7.70%	153	43.60%	171	48.70%	
	Degree of personal accomplishment							0.370
	Low degree	26	8.60%	135	44.90%	140	46.50%	
	Moderate degree	2	3.40%	24	40.70%	33	55.90%	
	High degree	2	5.90%	19	55.90%	13	38.20%	
Quality of life (mean, SD)	Level of physical	12.84	2.56	13.12	2.39	12.44	2.20	0.020
	Level of psychological	12.82	3.24	13.91	2.35	13.26	2.49	0.013
	Level of social	13.69	3.62	14.67	3.21	13.78	3.41	0.030
	Level of environment	13.70	2.44	14.37	2.60	14.17	2.67	0.408
Resilience	Low	6	6.4%	40	42.6%	48	51.1%	0.810
	Normal	24	8.2%	136	46.3%	134	45.6%	
	High	0	0.0%	2	33.3%	4	66.7%	

**Table 6:** Differences in burnout, quality of life, and resilience levels based on income level.

		≤ **RM 4,850**		**RM 4,850 – RM 10,959**		≥**RM 10,959**		***P*-value**
		***N***	**%**	***N***	**%**	***N***	**%**	
Burnout	Occupational exhaustion							0.859
	Low degree	25	71.40%	9	25.70%	1	2.90%	
	Moderate degree	147	73.90%	46	23.10%	6	3.00%	
	High degree	110	68.80%	45	28.10%	5	3.10%	
	Degree of depersonalization							0.87
	Low degree	4	80.00%	1	20.00%	0	0.00%	
	Moderate degree	27	71.10%	11	28.90%	0	0.00%	
	High degree	251	71.50%	88	25.10%	12	3.40%	
	Degree of personal accomplishment							0.959
	Low degree	214	71.10%	77	25.60%	10	3.30%	
	Moderate degree	43	72.90%	14	23.70%	2	3.40%	
	High degree	25	73.50%	9	26.50%	0	0.00%	
Quality of life (mean, SD)	Level of physical	12.82	2.39	12.66	2.24	12.81	1.71	0.844
	Level of psychological	13.49	2.58	13.52	2.33	14.28	2.44	0.567
	Level of social	14.12	3.27	14.25	3.67	14.89	2.53	0.713
	Level of environment	13.93	2.60	14.82	2.55	16.08	2.20	0.001
Resilience	Low	68	72.3%	24	25.5%	2	2.1%	0.001
	Normal	213	72.4%	74	25.2%	7	2.4%	
	High	1	16.7%	2	33.3%	3	50.0%	

Table 7 shows that burnout and resilience levels had no statistically significant differences between participants with different work duration. The only variable that showed statistically significant differences between different work duration was level of QoL. The mean score of physical health of participants who worked more than 10 h (11.38) was lower than participants who worked from 8 to 10 h (13.00) and participants who worked for 7 h daily (13.03; *p*-value < 0.001). The mean score of psychological health of participants who worked more than 10 h (12.35) was lower than participants who worked from 8 to 10 h (13.72) and participants who worked 7 h daily (13.68; *p*-value = 0.001). The mean score of social relationships of participants who worked more than 10 h (12.65) was lower than participants who worked from 8 to 10 h (14.38) and participants who worked 7 h daily (14.55), *p*-value = 0.003. The mean score of environment level in participants who worked more than 10 h (12.87) was lower than participants who worked 7 h daily (14.21) and participants who worked from 8 to 10 h (14.56; *p*-value < 0.001).

**Table 7:** Differences in burnout, quality of life, and resilience levels based on work duration.

		**Less than 7 h**		**7 h daily**		**From 8 to 10 h**		**More than 10 h**		***P*-value**
		***N***	**%**	***N***	**%**	***N***	**%**	***N***	**%**	
Burnout	Occupational exhaustion									0.368
	Low degree	0	0.00%	14	40.00%	18	51.40%	3	8.60%	
	Moderate degree	2	1.00%	64	32.20%	108	54.30%	25	12.60%	
	High degree	2	1.30%	39	24.40%	90	56.30%	29	18.10%	
	Degree of depersonalization									0.35
	Low degree	0	0.00%	2	40.00%	3	60.00%	0	0.00%	
	Moderate degree	1	2.60%	13	34.20%	22	57.90%	2	5.30%	
	High degree	3	0.90%	102	29.10%	191	54.40%	55	15.70%	
	Degree of personal accomplishment									0.188
	Moderate degree	4	1.30%	29.20%	29.20%	171	56.80%	38	12.60%	
	High degree	0	0.00%	15	25.40%	29	49.20%	15	25.40%	
	High degree	0	0.00%	14	41.20%	16	47.10%	4	11.80%	
Quality of life (mean, SD)	Level of physical	13.71	2.84	13.03	2.42	13.00	2.20	11.38	2.13	< 0.001
	Level of psychological	15.00	3.46	13.68	2.46	13.72	2.39	12.35	2.74	0.001
	Level of social	14.00	4.15	14.55	3.23	14.38	3.28	12.65	3.54	0.003
	Level of environment	15.38	1.31	14.21	2.46	14.56	2.57	12.87	2.80	< 0.001
Resilience	Low	1	1.1%	28	29.8%	45	47.9%	1	1.1%	0.325
	Normal	3	1.0%	88	29.9%	167	56.8%	3	1.0%	
	High	0	0.0%	1	16.7%	4	66.7%	0	0.0%	

Differences in burnout, quality of life, and resilience levels based on socializing duration were also measure in Table 8. No statistically significant differences were however found. Burnout, QoL, and resilience levels showed no statistically significant differences between HCWs who were infected and not infected with COVID-19 as shown in Table 9.

**Table 8:** Differences in burnout, quality of life, and resilience levels based on socializing duration.

		**No time**		**Less than 5 h**		**From 5 to 10 h**		**More than 10 h**		***P*-value**
		***N***	**%**	***N***	**%**	***N***	**%**	***N***	**%**	
Burnout	Occupational exhaustion									0.774
	Low degree	1	2.90%	7	20.00%	16	45.70%	11	31.40%	
	Moderate degree	6	3.00%	48	24.10%	61	30.70%	84	42.20%	
	High degree	5	3.10%	40	25.00%	52	32.50%	63	39.40%	
	Degree of depersonalization									0.292
	Low degree	1	20.00%	0	0.00%	1	20.00%	3	60.00%	
	Moderate degree	0	0.00%	12	31.60%	12	31.60%	14	36.80%	
	High degree	11	3.10%	83	23.60%	116	33.00%	141	40.20%	
	Degree of personal accomplishment									0.39
	Low degree	8	2.70%	70	23.30%	99	32.90%	124	41.20%	
	Moderate degree	1	1.70%	18	30.50%	20	33.90%	20	33.90%	
	High degree	3	8.80%	7	20.60%	10	29.40%	14	41.20%	
Quality of life (mean, SD)	Level of physical	12.43	2.89	12.66	2.38	12.77	2.30	12.89	2.29	0.833
	Level of psychological	12.44	3.59	13.38	2.58	13.58	2.43	13.64	2.45	0.410
	Level of social	13.33	4.02	13.59	3.28	14.34	3.45	14.46	3.25	0.161
	Level of environment	13.75	2.12	13.74	2.48	14.19	2.79	14.58	2.56	0.090
Resilience	Low	3	3.2%	25	26.6%	3	3.2%	25	26.6%	0.858
	Normal	9	3.1%	69	23.5%	9	3.1%	69	23.5%	
	High	0	0.0%	1	16.7%	0	0.0%	1	16.7%	

**Table 9:** Differences in burnout, quality of life, and resilience levels between health worker who were infected and not infected with COVID-19.

		**Not infected**		**Infected**		***P*-value**
		***N***	**%**	***N***	**%**	
Burnout	Occupational exhaustion					0.180
	Low degree	16	45.70%	19	54.30%	
	Moderate degree	102	51.30%	97	48.70%	
	High degree	95	59.40%	65	40.60%	
	Degree of depersonalization					0.830
	Low degree	2	40.00%	3	60.00%	
	Moderate degree	21	55.30%	17	44.70%	
	High degree	190	54.10%	161	45.90%	
	Degree of personal accomplishment					0.318
	Low degree	157	52.20%	144	47.80%	
	Moderate degree	34	57.60%	25	42.40%	
	High degree	22	64.70%	12	35.30%	
Quality of life (mean, SD)	Level of physical	12.76	2.44	12.80	2.20	0.865
	Level of psychological	13.63	2.49	13.39	2.54	0.341
	Level of social	14.19	3.26	14.16	3.47	0.924
	Level of environment	14.26	2.64	14.18	2.60	0.767
Resilience	Low	49	52.1%	45	47.9%	0.368
	Normal	159	54.1%	135	45.9%	
	High	5	83.3%	1	16.7%	

## Discussion

This study aimed to determine whether gender, specialty, age, education level, income level, work duration, duration of socialization, and COVID-19 infection status were associated with burnout, resilience, and QoL among HCWs. To summarize, the bivariate analyses found that in terms of resilience, there was a significant difference between males and females, and number of hours socializing. In terms of QoL, level of physical health was different in terms of specialty, age, education level, and work duration; levels of psychological and social health were associated with specialty, age, education level, and work duration; level of environment was associated with only specialty, age, and income level. No significant differences were found among the demographic categories in terms of burnout.

An interesting finding of our study was that more females reported low resilience compared to males. The results are in contrast with a study from the UK among HCWs, which found females to have a higher resilience level than males (50). Likewise, during the COVID-19 pandemic, female HCWs showed higher resilience in the domain of social support compared to males (51). However, a study among radiology workers from China showed that females had lower resilience scores than males (52). Moreover, a meta-analysis on gender and resilience found that males had higher resilience scores than females (53). According to Hirani et al. (54), females usually scored lower on resilience measures due to existing definitions of resilience not adequately reflect how various factors (e.g., gender roles, social factors, and the environment) interact to shape women's experience of and responses to facing difficulties in life (54).

Meanwhile, we found that a higher proportion among those who spent the highest number of hours socializing (>10 h) reported low resilience. It has been shown in a number of studies that social support is an integral aspect of resilience (55, 56). The results found in this study shows that the number of hours spent socializing may not equal social support, as social support includes elements such as a subjective perception of how much an individual is being supported by others (57). In contrast, an individual who spends high number of hours socializing (i.e. more than 10 h) may find that their energy and time are depleted due to excessive socializing. There is a need to further investigate in future studies the content of one's socialization activities which contributes to one's resilience.

QoL in terms of physical health, psychological health and social relationships of nurses was higher than doctors in this study. The results are not consistent with another study by Çelmeçe and Menekay (58), who found no difference in the QoL of nurses and doctors in Turkey during the COVID-19 pandemic (58). On the other hand, another study conducted in Spain during the COVID-19 pandemic found that primary care doctors had lower professional QoL compared to nurses (59). Doctors who are responsible for significant clinical decisions may report lower QoL due to assuming greater responsibilities during this period in time. In addition, according to Li et al. (60), doctors undergo more negative work-related experiences compared with nurses (60). These may be possible reason that the participant doctors suffered from lower QoL in all domains in this study, and it warrants further examination.

In terms of age, QoL of older HCWs were generally higher than that of younger HCWs in all aspects of QoL. The findings are consistent with another study on QoL among nurses in Saudi Arabia, where nurses who were older consistently reported higher QoL in comparison to those who were younger (<30 years old) (32). During the COVID-19 pandemic, a study among COVID-19 recovered HCWs in Bangladesh indicated that QoL was also higher among those with older age (61). Older age may be associated with higher QoL because increase in experience in professional work usually grows in tandem with age; greater experience at work has been proposed to be a factor leading to higher QoL (62).

We found an inverse relationship between participants' education level and QoL, where participants with a secondary education reported higher QoL in all aspects in comparison with participants with a tertiary education. Studies have reported that individuals with higher educational level reported higher QoL (63–65), possibly due to the beneficial influence of higher health literacy among those with more educational years (66). However, a study among HCWs in Malaysia during the COVID-19 pandemic showed that there were no differences in all QoL domains in terms of education level (67). In our study, individuals with a tertiary education may have lower QoL due to assuming responsibilities and making decisions which would more directly impact patients, in comparison with those who had secondary education. The greater responsibility assumed in patient healthcare may have contributed to lower QoL due to experiencing more job-related stress.

All domains of QoL were lower among HCWs who worked more than 10 h per day. To cope with the rising demands for healthcare during the COVID-19 pandemic, accompanied by the depletion of HCWs due to infection of COVID-19 virus, there were a number of strategies used to increase HCW capacity (68). The most common strategy reported was extending the working hours of HCWs, and this includes working overtime, canceling leaves, and allowing back-to-back shifts (69). The length of working hours among HCWs has been associated with a number of negative physical, psychological, and safety outcomes among HCWs, such as musculoskeletal pain (70), fatigue and isolation (71), and less time to participate in social activities (69). All this may have negatively affected the QoL of the HCWs who worked for longer hours.

In this study, HCWs who earned <RM 4,850 reported lower QoL in their environmental level in comparison with those who earned more per month. The results are consistent with past studies, in which individuals from a lower-income background may suffer from lower environment QoL (61, 72–74). For example, a large-scale survey in Malaysia with 18,607 rural residents found that compared with individuals with low income, those enjoying middle and high income had higher perceived QoL in all four domains (75). Environmental QoL may be lower among HCWs with low income due to poorer living conditions, financial issues, and physical insecurities (61, 73).

The study results showed that there were no differences in terms of burnout, resilience, and QoL among HCWs who had been infected or not infected by the COVID-19 virus. The results are not consistent with extant literature, where those who had been infected by the COVID-19 virus reported higher burnout levels (36, 76). Infection status may also lead to physical and psychological ramifications, eventually leading to lower QoL (35). However, a study conducted among the Italian general population found that COVID-19 infection did not significantly predict resilience (77). As this study was carried out during the later stages of the COVID-19 pandemic when most HCWs had been vaccinated against the virus, having been infected by the virus may not have affected the HCWs in terms of their burnout, resilience, and QoL.

The results of this study have significance on identifying HCWs who may be at risk of burnout, low resilience, and low QoL during the COVID-19 pandemic and beyond. It is important to identify the related demographic and work factors in order to more effectively screen HCWs for the presence of these conditions, and to provide age-, gender-, and specialty-appropriate interventions. Healthcare authorities should be mindful of the negative consequences of long working hours on HCW's QoL. Attention should be paid to younger HCWs' and doctors' QoL, to find out the specific work, physical or psychological characteristics contributing to lower QoL. Issues pertaining to the environmental health of HCWs with lower income should also be identified and addressed.

This study has a few limitations. Being a cross-sectional study, we could not infer a cause-and-effect relationship between the variables. Since the participants were not randomly sampled, we could not rule out the presence of bias in this study. Other aspects which possibly contribute to burnout, resilience, and QoL among HCWs were not explored, such as the influence of religion, workload, and psychological distress. Future studies can be conducted to understand the role of resilience and coping in mediating burnout and QoL among HCWs. A combination of quantitative and qualitative methodology in future studies would be able to provide in-depth information on the possible causes that led to our findings.

## Conclusion

This study explored demographic and work-related factors associated with burnout, resilience, and QoL among HCWs in Malaysia during the COVID-19 pandemic. The findings of this study call out for some specific interventions from policy makers. Nurses were more prone to report poor scores on burnout and resilience than doctors. However, they reported higher mean score on various dimensions of QoL than doctors. Age of the HCWs was an important factor in determining resilience as HCWs with higher age reported high resilience. Though inferential but low age of the HCWs means they are new to the profession and therefore overburdened and not experienced as compared to their senior counterparts who have years of experience, that works as a coping strategy for them. The study confirmed that higher income level leads to better resilience and longer work duration leads to low level of QoL. It is imperative that healthcare practitioners and policy makers adopt and implement interventions to promote a healthy workplace environment, address ethical concerns, and prevent burnout among healthcare workers during and beyond the COVID-19 pandemic. Managing the issue of long working hours could possibly result in improved resilience, burnout, and QoL among HCWs. Though this study may be able to inform some policy specific areas where interventions are needed, identifying effective solutions and evaluating their effectiveness will require larger and interventional studies.

## Data availability statement

The raw data supporting the conclusions of this article will be made available by the authors, without undue reservation.

## Ethics statement

The studies involving human participants were reviewed and approved by Ethics Committee of Management and Science University. The patients/participants provided their written informed consent to participate in this study.

## Author contributions

All authors made a significant contribution to the work reported, whether that is in the conception, study design, execution, acquisition of data, analysis and interpretation, or in all these areas, took part in drafting, revising, or critically reviewing the article, gave final approval of the version to be published, have agreed on the journal to which the article has been submitted, and agree to be accountable for all aspects of the work.
